# Effects of changes in the waveform and frequency of radio frequency energy on tissue ablation range

**DOI:** 10.1371/journal.pone.0308691

**Published:** 2024-09-19

**Authors:** Jinsu An, Dong-Sung Won, Yubeen Park, Jung-Hoon Park, Ki-Hyeon Park, Ji-Ho Lee, Hyung-Sik Kim

**Affiliations:** 1 Department of Biomedical Engineering, School of ICT Convergence Engineering, College of Science & Technology, Konkuk University, Chungju-si, Chungcheongbuk-do, Republic of Korea; 2 Biomedical Engineering Research Center, Asan Institute for Life Sciences, Asan Medical Center, Songpa-gu, Seoul, Republic of Korea; 3 Department of Gastroenterology, Asan Medical Center, University of Ulsan College of Medicine, Songpa-gu, Seoul, Republic of Korea; 4 Department of Convergence Medicine, Asan Medical Center, University of Ulsan College of Medicine, Songpa-gu, Seoul, Republic of Korea; 5 Department of Mechatronics Engineering, School of ICT Convergence Engineering, College of Science & Technology, Konkuk University, Chungju-si, Chungcheongbuk-do, Republic of Korea; Universidad Tecnica de Ambato, ECUADOR

## Abstract

This study reports the effects of changes in the waveform and frequency of radio frequency (RF) energy on the tissue ablation range. We developed a 70-watt RFA generator that provides sine and square waves and allows frequency control between 10 Hz and 500 kHz. The changes in the ablation range according to the waveform and frequency were observed using the developed generator. In the waveform variation test, the distance between the electrodes and the electrode type were changed for both waveforms with the frequency set to 500 kHz. In the frequency variation test, the waveform and electrode type were changed with the frequency set to 10, 100, and 500 kHz, while the distance between the electrodes was set to 20 mm. A fixed 45 voltage was applied using the bipolar method. RF energy was applied for 90 s in vitro. The temperature was regulated to not exceed 70°C. The ablation range was calculated using ImageJ software. The analysis results showed that the ablation range was larger with the square wave than with the sine wave and at 10 kHz than at 500 kHz. The developed generator can advance research on ablation area and depth in RF ablation.

## 1. Introduction

Radio frequency ablation (RFA) is a minimally invasive therapy that causes coagulative necrosis of tissues by increasing their temperature using a high-frequency current [1, 2]. RFA is used as a nonsurgical treatment for patients who cannot undergo surgical treatment. It has few complications, requires a short hospitalization period, and is also used to treat hypovascular and fibrous metastatic cancers with low treatment effects [3, 4]. When a high-frequency current is applied to tissues through electrodes inserted into the body, the ions in the tissues are arranged in the same direction as that of the alternating current and vibrate along the direction of the current. Thus, frictional or resistance heat is generated in the tissues, making the tissue around the electrodes serve as the heat source for RFA [1]. Various types of living tissues have different structures and properties, such as hydration, density, and impedance [5, 6]. Therefore, the size of the tissue ablated by RFA could vary based on tissue properties, output power of RF energy, application time, tissue temperature, and the electrodes used [1, 7–9].

Many studies have been conducted to increase the ablation size while changing the variables affecting RFA. For example, studies have been conducted to control the ablation range while changing the amount of electrical energy applied to living tissues. Generally, the higher the power, the higher the current flowing through the tissues, and the larger the ablation range [9, 10]. However, the increase in the ablation range was limited even with increased power owing to the shape of the electrode that delivered energy [8, 9]. Moreover, when the impedance of the current flow path increased, the ablation range did not increase even with increased power [7, 11]. An experiment to check the ablation range while changing the RF energy application time was also performed. Although there were differences in the tissue properties and power over a duration of approximately 8 min, the ablation range increased with ablation time; however, the changes were minimal after this duration [12–15]. Furthermore, when energy was applied for a long time at a high tissue temperature, the tissues were carbonized as moisture evaporated. Consequently, the impedance increased significantly, making it impossible to deliver energy [16, 17]. To overcome this problem, a cooling method that supplies saline through electrodes or temperature control using temperature sensors attached to electrodes has been reported [16–18]. Studies have also been conducted to expand the ablation range by enlarging the electrode size to an umbrella or balloon shape. The ablation range increased with the electrode and tissue contact areas. However, the shape of the electrodes limited available tissues [8, 19, 20]. Previous studies have focused on electrical energy, time, and electrode shape. However, in most clinical and research studies, the RF energy applied in a high-frequency generator was only a sine wave, and only one fixed frequency was used among the frequencies from 400 kHz to 500 kHz. A study on the ablation range based on changes in the waveform and frequency of electrical energy is necessary because many types and different characteristics of tissues, such as their hydration, density structure and composition. Recently, studies related to waveforms and frequencies have predominantly utilized theoretical approaches, with a focus on finite element analysis and simulation studies [21, 22]. Theoretically, a square wave has higher energy than a sine wave at the same amplitude and frequency. Additionally, at lower frequencies, the energy transmission distance increases. We hypothesized that using a square wave and lower frequencies could increase the ablation range compared to the conventional method. Therefore, this study developed an RFA generator that can control the waveform and frequency. Variations in the ablation range of the tissues were observed according to the waveform, frequency, electrode type, and distance. An in vitro experiment was performed using pork as tissues, with the tissue temperature not exceeding 70°C.

## 2. Method

### 2.1 Development of radio frequency generator

**Fig 1:**
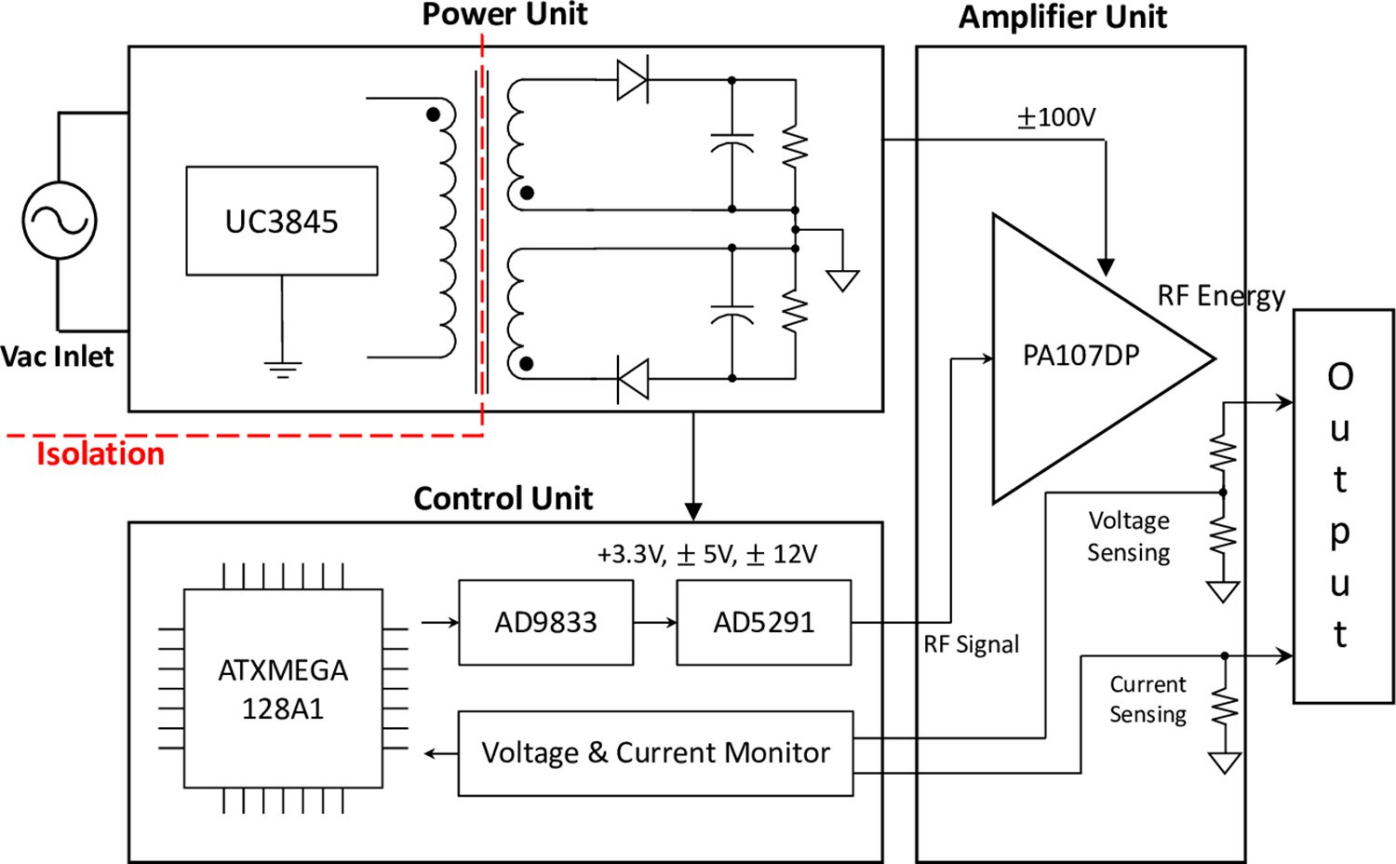
Block diagram of the developed radio frequency generator.

Fig 1 shows a block diagram of the RFA generator. The RFA generator comprised power, control, and amplification units.

For the power unit, a flyback topology capable of electrical isolation while converting the AC inlet to DC was used. It was designed to output up to 200 watts with a 100 V DC voltage using UC3845 (STMicroelectronics, Geneve, Switzerland), a current-mode pulse-width modulation (PWM) controller. For the control unit, a microcontroller ATXMEGA128A1 (Microchip Technology, Inc., Chandler, AZ, USA) was used to control the overall system operations and RF signals while monitoring the impedance and temperature in real-time. RF signals that can convert sine or square waves to frequencies in the range of 10 kHz–500 kHz were generated using an AD9833 programmable waveform generator (Analog Devices, Inc., Wilmington, MA). The amplitudes of the RF signals generated using a digital potentiometer AD5291 (Analog Devices, Inc., Wilmington, MA) were controlled. The output parameters (waveform, frequency, and power) can be fine-tuned because all three control variables can be controlled digitally. The generated RF signals were delivered to the amplification unit before being outputted. A high-voltage power-operational amplifier PA107DP (Apex Microtechnology, Tucson, AZ, USA) was used for the amplification unit. The maximum output voltage, maximum output current, and output frequency range of this amplifier were 90 V, 1.5 A, and 2 MHz, respectively. Various waveforms and frequencies can be output even with a simple structure because a monolithic operational amplifier was used. An output of up to 70 watts can be achieved using this method. Maintenance of the set output power was regulated in real-time using a sense resistor and voltage divider comprising resistors.

An analog front-end ADS1292 (Texas Instruments, Inc., Dallas, TX, USA) was used for measuring the biopotential. The impedance changes and contact between the tissue and electrode were measured before and after applying RF energy. The temperature was monitored in real-time using an instrumentation amplifier AD620 (Analog Devices, Inc., Wilmington, MA) with four possible temperature settings (60, 70, 80, and 90°C). The selected temperature was maintained by setting the RF energy output on and off. The five control parameters of the RFA generator (output power, waveform, frequency, temperature, and time) can be set using a user interface comprising a character liquid crystal display (LCD), buttons, and a rotary encoder.

### 2.2 Experimental protocol

**Fig 2:**
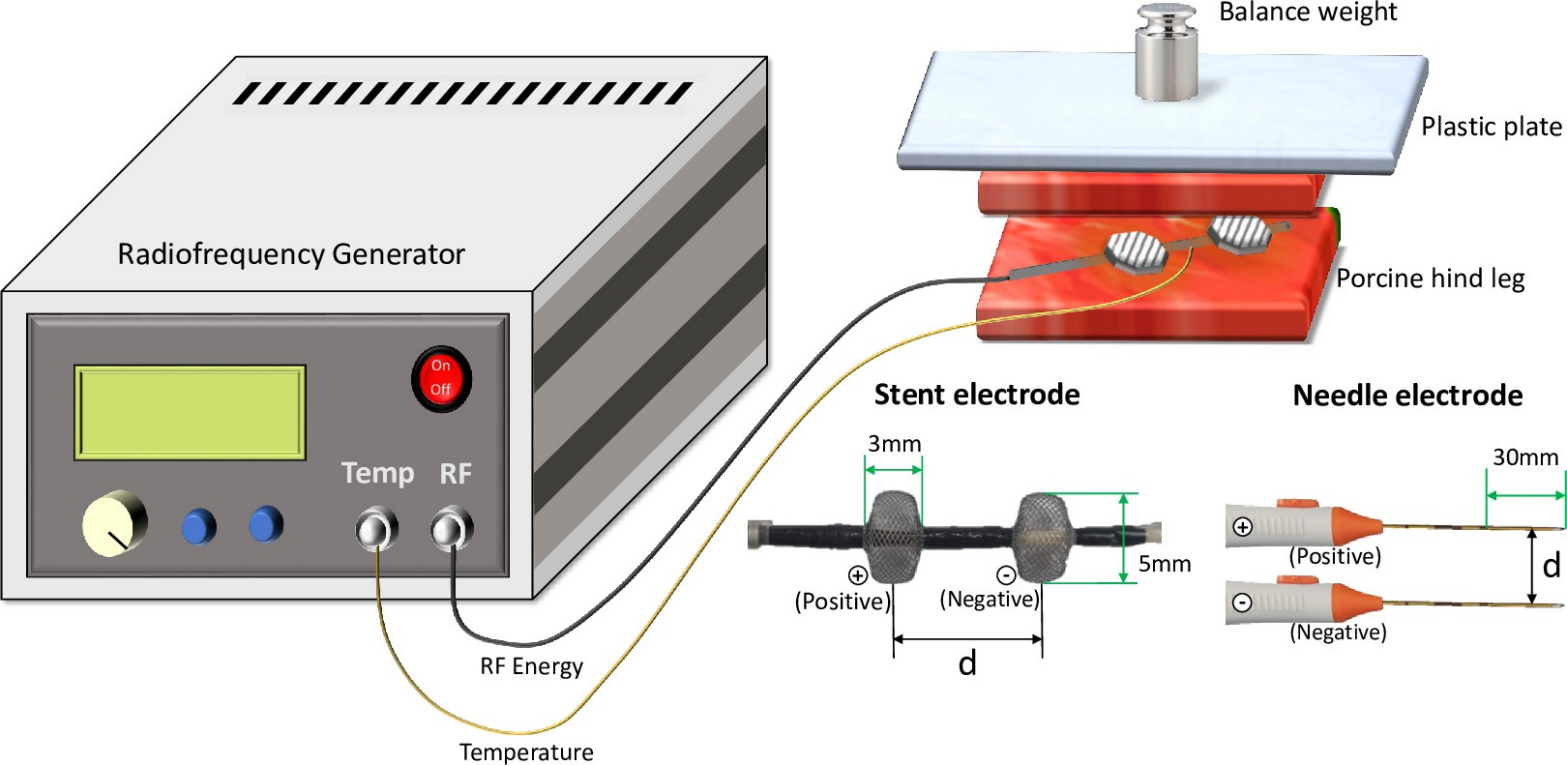
Overall experimental setup and two types of electrodes used in this study: Stent and needle electrodes.

Fig 2 shows the experimental setup. Changes in the tissue ablation range due to the output waveform and frequency were investigated using the developed RFA generator. Furthermore, changes in the ablation range according to the control parameters were observed while changing the electrode type and distance between the electrodes.

Refrigerated pork hind legs supplied by a butcher shop were used in the in vitro experiment. To maintain a constant distance (d) between the electrodes and insertion depth between experiments, 40 mm-sliced tissues were cut in half and divided into two A sandwich-shaped experimental sample was set up by positioning bipolar electrodes at the center of one piece of tissue and covering it with the other piece. The stent electrode was used in a deployed state. A plastic plate larger than the sample was placed on top of the sample to maintain a constant contact area between the electrode and tissue. It was then adjusted to the same impedance using a balanced weight. For the stent electrode, the electrode distance was set to approximately 90.3 Ω ± 3.6 Ω at 10 mm, 101.5 Ω ± 2.9 Ω at 15 mm, and 112.5 Ω ± 6.8 Ω at 20 mm. The resistance of the needle electrode was set to approximately 62.3 Ω ± 2.5 Ω at 10 mm, 72.5 Ω ± 6.2 Ω at 15 mm, and approximately 81.4 Ω ± 3.8 Ω at 20 mm. Furthermore, the temperature sensor was placed in the center between all of the two types of electrodes, and samples within an initial temperature of approximately 15°C ± 3°C were maintained for the experiments. All experiments were performed by applying RF energy for 90 s, with the average temperature not exceeding 70°C.

Changes in the ablation range caused by the output waveform were tested using sine and square waves. The output voltage and frequency were fixed at 45 V and 500 kHz, respectively. The electrode distance (d), based on the distance between the electrode centers, was varied to 10, 15, or 20 mm during the experiment. A bipolar stent electrode (S&G Biotech, Ltd., Yongin, South Korea) [10] was used for the prototype. An Octopus RF electrode (STARmed, Ltd., Goyang, South Korea) was used for the bipolar needle electrode. An experiment on the change in ablation range according to the output range was performed for both waveforms at 10, 100, and 500 kHz. The output voltage was fixed at ±45 V, and the largest distance of 20 mm was applied for both types of electrodes.

### 2.3 Data analysis

Twelve experiments were performed for the output waveform and frequency. A total of 72 experiments were performed using three samples for each experimental condition. The surface parallel to the electrodes of the samples is called the ablation area, the surface perpendicular to the electrodes is called the ablation depth, and the sum of the area and depth is defined as the range. The ablation area and depth of the samples were quantified using ImageJ software (National Institutes of Health, Bethesda, MD, USA), and the average was calculated. Statistical analysis was performed to detect the differences among the ablation range groups. ANOVA and t-test were performed using SPSS software (SPSS 29.0.2, IBM, Chicago, IL). Data were expressed as a mean ± standard deviation (M±SD). P<0.05 was considered statistically significant.

## 3. Results

### 3.1 Testing the output of the radio frequency generator

**Fig 3:**
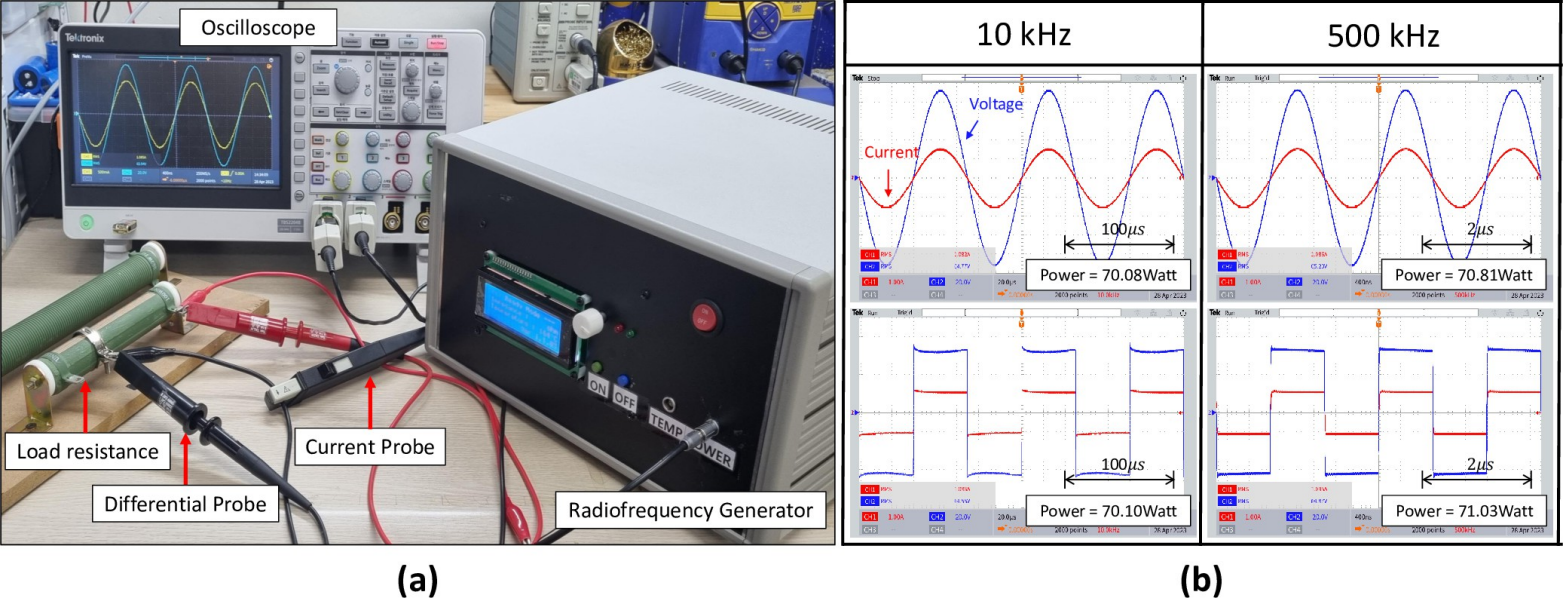
Operation test of the developed radio frequency generator. (a) Actual appearance of the generator and operation test environment, (b) Maximum output of the sine and square waves for frequencies of 10 kHz and 500 kHz.

Fig 3 shows the actual view and operational results of the RFA generator. A load resistance of 60 Ω was applied under maximum output conditions. The voltage and current of the RF waveform were measured using a TBS200B oscilloscope (Tektronix, Inc., Beaverton, OR), high-voltage differential probe P5210A (Tektronix, Inc.), and current amplifier TCPA300 (Tektronix, Inc.). The effective value was used for the power and calculated using *P* = *V_rms_*×*I_rms_*. Up to 70 watts was outputted with frequencies of 10 kHz to 500 kHz for both waveforms.

### 3.2 Tissue ablation according to the waveform

**Fig 4:**
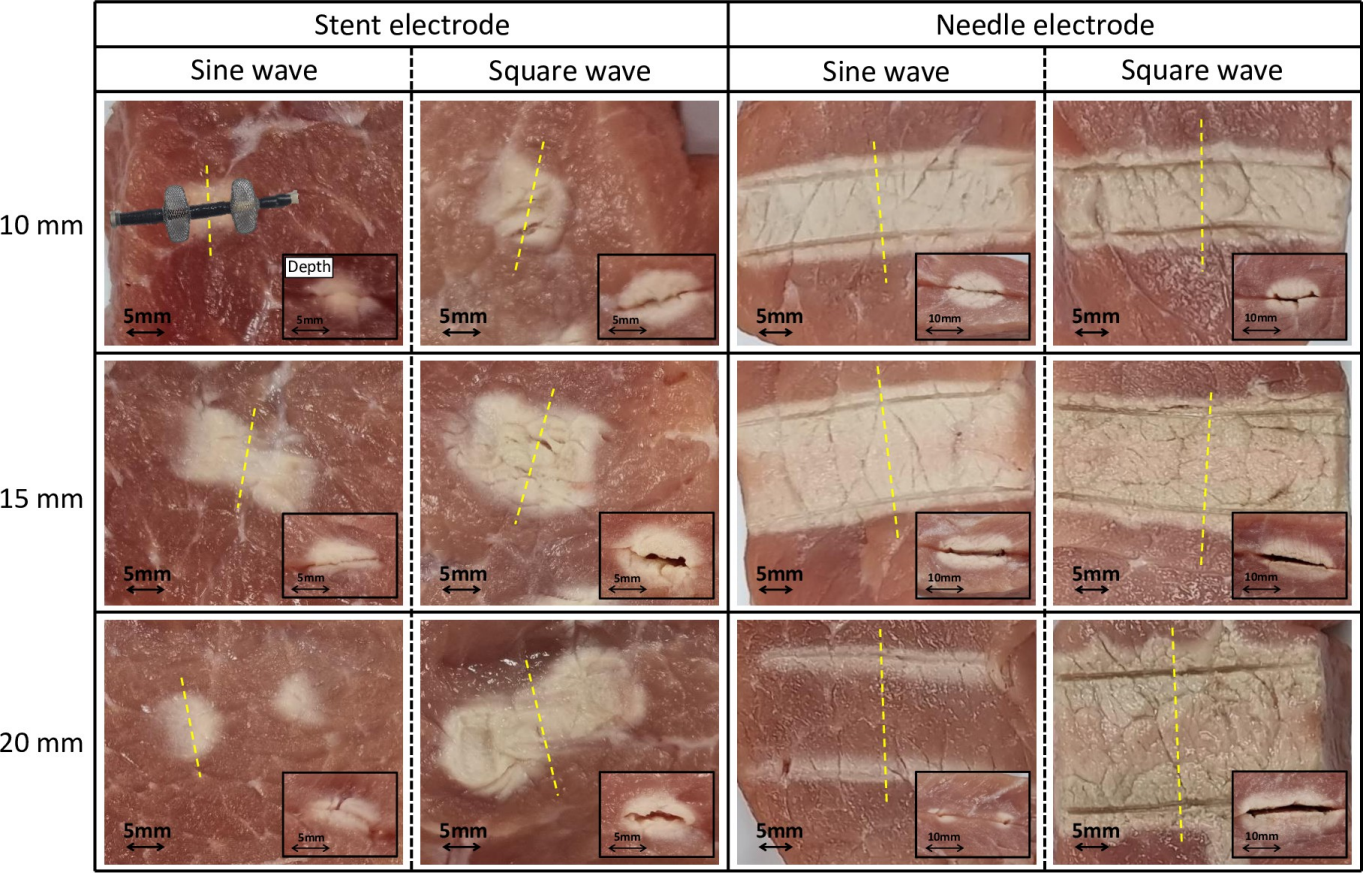
Changes in waveform and ablation range according to each experimental condition. The large photograph at the center shows the ablation area. The small photograph at bottom right shows the ablation depth. The stent electrode was positioned as shown in the first photograph.

Fig 4 shows the changes in the waveform, ablation area, and depth under each experimental condition.

In the experiment using the stent electrode, a sine wave was ablated to the electrode center at 10 mm and 15 mm. The temperature was maintained after the set maximum temperature of 70°C was reached. By contrast, the surroundings of the electrode were ablated at 20 mm, but not the electrode center. The maximum temperature at the center was approximately 44°C. The square wave was ablated to the electrode center at every distance. The ablation area increased with the distance, but the ablation depth decreased. The temperature at the center increased to 70°C and remained stable. In the experiment using the needle electrode, a sine wave was ablated to the center at 10 mm and 15 mm. At 20 mm, only the surroundings of the electrode were ablated, and the maximum temperature at the center was approximately 40°C. The square wave was ablated to the center under all conditions. The ablation area and depth increased with the distance. The center temperature also increased to 70°C.

Table 1 lists the mean and standard deviation ablation ranges (M±SD) calculated for the two waveforms according to the electrode type and distance. Samples with only the surroundings of the electrode ablated were excluded from the analysis. In experiments using the stent electrode, at an electrode distance of 10 mm, the square wave produced a significantly larger ablation area than the sine wave, with the sine wave producing an area of 91.3±8.4 mm^2^ and the square wave producing an area of 165.5±10.2 mm^2^ (p<0.001). At 15 mm, the sine wave produced an area of 222.5±14.1 mm^2^, while the square wave produced an area of 353.8±19.2 mm^2^ (p<0.001). In experiments using the needle electrode, at an electrode distance of 10 mm, the square wave produced an ablation area of 396.7±10.4 mm^2^, while the sine wave produced an area of 372.7±12.4 mm^2^. At 15 mm, the square wave produced an area of 517.7±20.4 mm^2^, while the sine wave produced an area of 485.0±17.9 mm^2^. In two conditions, the square wave produced a slightly larger area, but it were not statistically significant (p>0.05). The ablation depth showed significant differences under all four conditions (p<0.05). At an electrode distance of 20 mm, which was excluded from the analysis, the sine wave did not form a continuous ablation area, whereas the square wave formed a sufficient ablation area. The needle electrode exhibited a larger ablation area and depth than the stent electrode under all experimental conditions.

**Table 1:** Two waveforms and ablation range according to electrode type and distance (M±SD, unit: mm^2^).

Ablation condition			10 mm	15 mm	20 mm
Ablation Area	Stent	Sine	91.3±8.4	222.5±14.1	-
		Square	165.5±10.2	353.8±19.2	428.5±22.0
	Needle	Sine	396.7±10.4	484.9±17.9	-
		Square	372.7±12.4	517.7±20.4	681.8±28.8
Ablation Depth	Stent	Sine	73.9±2.4	71.6±14.6	-
		Square	111.5±2.8	107.5±9.1	78.6±8.6
	Needle	Sine	154.6±2.8	201.9±9.4	-
		Square	133.0±3.1	221.6±7.9	351.4±10.1

**Fig 5:**
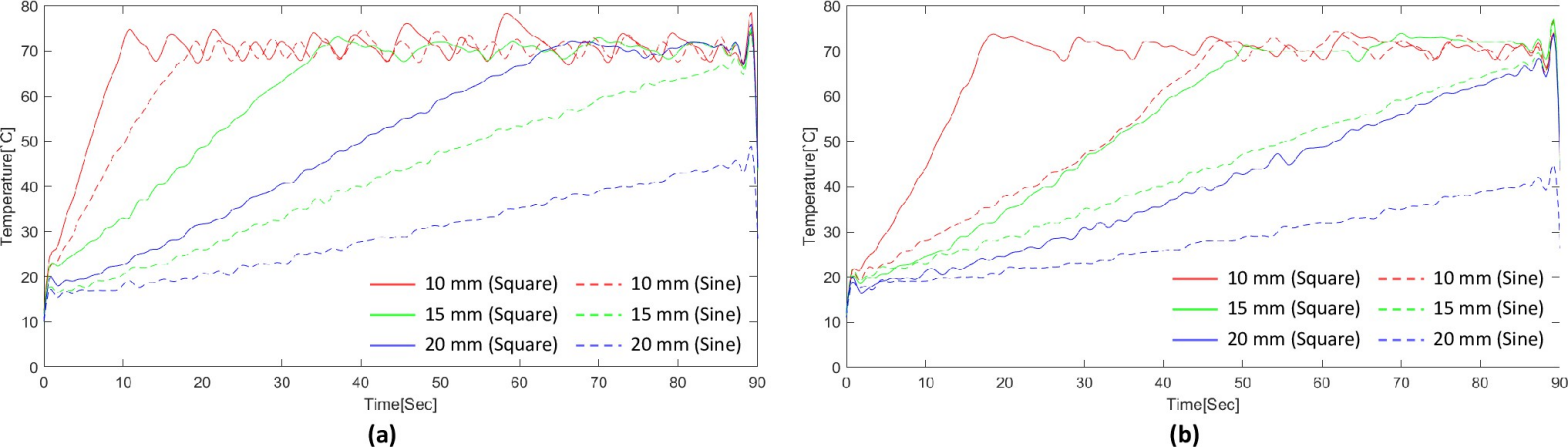
Changes in waveform and temperature rise according to each experimental condition. (a) Temperature rise graph of the experiment using the stent electrode, (b) Temperature rise graph of the experiment using the needle electrode.

Fig 5 shows the electrode type and temperature profile by distance based on the waveform changes. The temperature rise time varied according to the RF waveform, electrode type, and electrode distance. The square wave reached the set temperature faster than the sine wave. The stent electrode exhibited a faster temperature increase than the needle electrode. In addition, the shorter the electrode distance, the higher the temperature.

### 3.3 Tissue ablation according to the frequency

**Fig 6:**
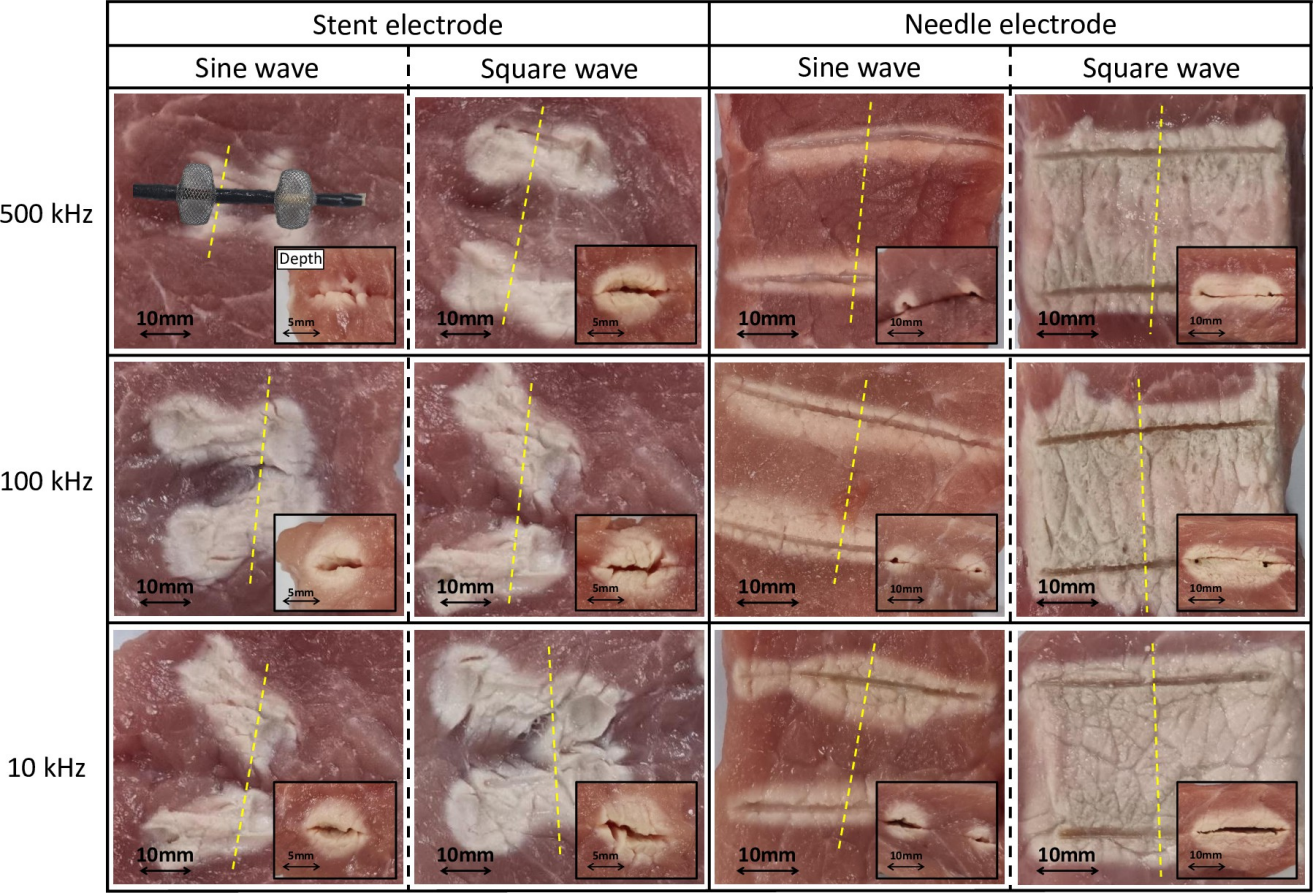
Ablation range according to frequency change and each experimental condition. The large photograph at the center shows the ablation area. The small photograph at bottom right shows the ablation depth. The stent electrode was positioned as shown in the first photograph.

Fig 6 shows the mean and standard deviation ablation area and depth (M±SD) with varying frequencies under each experimental condition.

In the experiment using the stent electrode, the sine and square waves exhibited a larger ablation area and depth and a faster temperature rise as the frequency decreased from 500 kHz to 100 Hz and 10 Hz. The center temperature of the square wave increased to 70°C, and every sample was ablated. A sine wave was ablated only around the electrode at a 500 kHz frequency. In the experiment using the needle electrode, a sine wave was ablated only in the surroundings of the electrode at all three frequencies. However, a square wave was ablated at the center of the electrode. Both waveforms showed an increased ablation area and depth as the frequency decreased.

Table 2 shows the ablation ranges calculated using the three frequencies and waveforms and according to electrode type. Samples that were not ablated to the tissue center were excluded from the analysis. In experiments using the stent electrode, the sine wave ablated a larger area at 100 kHz and 10 kHz compared to 500 kHz (p<0.01), but there was no statistically significant difference between 100 kHz and 10 kHz (p = 0.52). For the square wave, a larger ablation area was formed as the frequency decreased (p<0.01). In experiments using the needle electrode, the sine wave only ablated the area around the electrode and was thus excluded from the analysis. However, it was observed that the ablated thickness increased as the frequency decreased. For the square wave, the ablation area increased as the frequency decreased (p<0.05). In the frequency change experiment, the needle electrode exhibited a larger ablation area and depth than the stent electrode under all experimental conditions.

**Table 2:** Ablation ranges according to three frequencies, waveforms, and electrode type (M±SD, unit: mm^2^).

Ablation condition			500 kHz	100 kHz	10 kHz
Ablation Area	Stent	Sine	-	369.1±12.5	374.0±13.5
		Square	405.0±8.7	403.3±4.7	479.6±12.4
	Needle	Sine	-	-	-
		Square	625.3±6.2	630.9±7.4	641.8±8.8
Ablation Depth	Stent	Sine	-	67.2±6.1	75.4±4.9
		Square	84.5±7.6	107.5±10.8	115.7±18.6
	Needle	Sine	-	-	-
		Square	347.4±12.5	419.5±21.8	448.3±22.4

**Fig 7:**
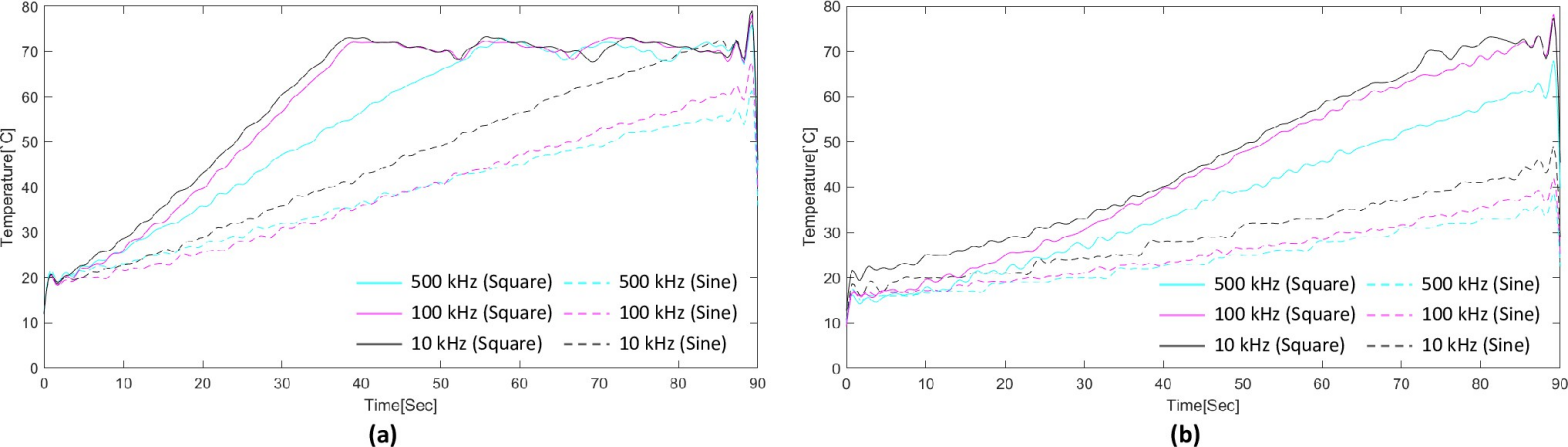
Temperature rise according to changes in frequency and each experimental condition. (a) Temperature rise graph of the experiment using the stent electrode, (b) Temperature rise graph of the experiment using the needle electrode.

Fig 7 shows the temperature profiles by electrode type and waveform based on the frequency changes. Similar to the waveform change experiment, as the frequency decreased, the temperature increased faster as the frequency decreased with the square wave than with the sine wave and with the stent electrode than with the needle electrode.

## 4. Discussion

This study reports the effects of the waveform and frequency of RF energy on the tissue ablation range. In this study, we developed an RFA generator that can provide sine and square waves and allow frequency control between 10 Hz and 500 kHz. The experiments with varying waveform, frequency, electrode type, and distance between electrodes revealed that the ablation area and depth were the largest for the square wave and low frequency. The needle electrode formed a larger ablation range than the stent electrode because it has a wider contact area with the tissue.

The developed RFA generator has the following advantages: first, the RF energies of the various waveforms were generated. In addition to the sine and square waves used in this study, various other waveforms, amplitudes, and frequency modulations are possible. Thus, the findings of this study can be applied to research on increasing ablation efficiency for various types of tissues; second, the output impedance was small. The impedance of the tissue, which was the load of the RFA generator, varied according to type [5, 6]. When the generator output impedance was high, the current decreased at the same power, and the ablation range decreased [11]. Thus, low-power impedance can effectively deliver energy to various tissues; third, RF energy can be output at various frequencies. Most RFA generators use transformers to facilitate output control and galvanic isolation [23–25]. The tissue impedance also changes with the frequency [5]. When a transformer is used, the output frequency is fixed, and the ablation result differs, even when the same energy is applied. The variability in the ablation range can be reduced by matching the RF energy of the frequency with the impedance measurement.

In the experiment on waveform changes, the ablation range increased more in the square wave compared to that in the sine wave. The same peak voltage of ±45 V was applied to both waveforms; however, the flow current was higher for the square wave than for the sine wave. This was because when the effective current for the square wave was 1, the effective current for the sine wave was approximately 0.7 [26]. The mean effective currents of the square and sine waves used in the experiment were 0.92 A and 0.64 A, respectively, corresponding to a difference of approximately 0.696 times. The tissue temperature reached 70°C faster in the square wave than in the sine wave owing to the high current. The impedance decreased when the tissue temperature increased [17, 27]. The high current flowed easily owing to the decreased impedance, resulting in the ablation of the center even with increased distance between the electrodes [7, 11]. In the experiment on frequency changes, the ablation range increased with low-frequency RF energy compared to that with high-frequency RF energy. In skin-depth studies of biological tissues, the energy transfer depth increased as the frequency decreased [28, 29]. In this study, the mean effective current increased from 0.86 A to 1.06 A and 1.12 A as the frequency decreased from 500 kHz to 100 kHz and 10 Hz. High current enables a rapid temperature rise rate and a large ablation range. These results suggest that variations in waveform and frequency enable the control of the rate of temperature increase, thereby allowing for the adjustment of the ablation range.

The ablation range differed according to the electrode type. Under the same conditions, the needle electrode showed a larger ablation range than the stent electrode. This result was attributed to the difference in the contact area between the two types of electrodes and the tissues. The larger the contact area, the smaller the impedance, and the faster the current flow. The mean impedances of the tissue measured using each electrode were 102 Ω and 72 Ω for the stent and needle electrodes, respectively. More current flowed into the needle electrode owing to the lower impedance.

This study has several limitations. It was conducted exclusively on the muscle tissues of the pork hind legs, chosen for the accessibility of sample acquisition and the clarity in identifying the ablation range. However, it is necessary to study the variations in ablation range based on waveform and frequency changes in tissues such as the liver, lungs, and bones where RFA is clinically applied. Moreover, these experiments were performed only in an in vitro environment. The tissue activities of the cells were suspended in this study; therefore, additional ex vivo and in vivo experiments are necessary. Research on the side effects of tissue caused by waveform and frequency is also essential. For example, the use of low frequencies in cardiac ablation is limited because it can induce arrhythmias [30, 31]. Such complication can significantly impact the patient’s safety. Therefore, it is crucial to thoroughly research and understand the potential side effects that may occur under various frequency conditions. This understanding will help provide more safer and better effective treatment methods for patients in the context of using RFA treatment.

## 5. Conclusion

In this study, we hypothesized that using a square wave, which has higher energy than a sine wave, and a lower frequency, which can transmit energy further, would increase the ablation range. To verify this, we developed a radiofrequency (RFA) generator capable of adjusting the waveform and frequency and observed changes in the ablation range according to the various electrical characteristics of RF energy. Traditional RF ablation using sine waves required higher voltage to achieve a wide ablation range. However, it was confirmed that by adjusting the frequency and waveform, a wide ablation range could be obtained with control of the temperature rise rate at the same voltage. This suggests that using various waveform and frequency changes can provide new methods for expanding and controlling the size and depth of the ablation range.
